# The incidence of congenital hypothyroidism and its determinants from 2012 to 2014 in Shadegan, Iran: a case-control study

**DOI:** 10.4178/epih.e2016021

**Published:** 2016-05-26

**Authors:** Ehsan Keshavarzian, Ali Asghar Valipoor, Mohammad Reza Maracy

**Affiliations:** 1 Student Research Center, School of Health, Isfahan University of Medical Sciences, Isfahan, Iran; 2 Abadan School of Medical Sciences, Abadan, Iran; 3 Department of Epidemiology and Biostatistics, School of Public Health, Isfahan University of Medical Sciences, Isfahan, Iran

**Keywords:** Incidence, Hypothyroidism, Live birth, Case-control studies, Risk factors

## Abstract

**OBJECTIVES:**

Congenital hypothyroidism (CH) is one of the major causes of preventable mental retardation in infants. The aim of this study was to determine the incidence of CH in Shadegan, Khuzestan Province, Iran from 2012 to 2014 and to identify the risk factors associated with CH.

**METHODS:**

A total of 203 cases were confirmed from 2012 to 2014 in Shadegan, with 66, 86, and 51 patients reported in 2012, 2013, and 2014, respectively. A total of 3,900, 3,991, and 4,050 live births occurred in 2012, 2013, and 2014, respectively. The controls (n=657) were selected using a random number table, and a case-control study was carried out to determine the risk factors for neonatal CH, including demographic, environmental, and medical factors.

**RESULTS:**

The incidence of CH was 17.0 per 1,000 live births in 2012, 21.5 per 1,000 live births in 2013, and 12.6 per 1,000 live births in 2014. This study showed that the likelihood of CH in children born to parents with a history of consanguineous marriage was 2.41 times greater than in children born to parents with no such history (odds ratio, 2.41; 95% confidence interval [CI], 1.65 to 3.53). This study also found that CH was 3.4 times more likely (95% CI, 2.29 to 5.20) in infants born in urban settings than in infants born in rural areas.

**CONCLUSIONS:**

The incidence of CH in Shadegan from 2012 to 2014 was approximately 17 times greater than the expected incidence in Iran. CH was associated with a history of consanguineous marriage and urbanization.

## INTRODUCTION

Hypothyroidism is defined as the presence of a thyroid-stimulating hormone level of more than 10 mU/L in combination with a thyroxin (T4) level of less than 6.5 mU/L [1]. CH is a common cause of preventable mental retardation [2]. In general, infants with CH are slow to grow and develop, their voice is hoarse, and they exhibit delays learning to speak. The degree of physical and mental retardation in these patients increases with their age. Moreover, sexual development can be delayed or never happen [3]. The incidence of CH is one per 4,500 live births in the US, one per 3,000 live births in Europe, and one per 5,700 live births in Japan [4]. In Greece, it is one per 800 live births [5]. Some studies have investigated the incidence of neonatal CH in Iran, finding the incidence of CH in Iran to be very high [6-8]. According to research conducted in Isfahan, Tehran, and Shiraz, the average incidence of CH was determined to be one per 1,000 live births. The greatest incidence of CH has been reported to occur in the fall and winter [9,10]. In another study conducted in Isfahan, the maximum incidence of the disease was found to be in August [11]. Another study conducted to assess the risk factors for CH showed that many factors contribute to the incidence of this disease [12]. The risk of CH can be increased by taking certain medications during pregnancy, such as amiodarone, cytokines, dopamine, drugs containing iodine, lithium, phenytoin (Dilantin), rifampicin, and steroids [13]. Infants weighing less than 2,000 g have a twice greater likelihood of developing CH than other infants. Additionally, the risk of CH in infants weighing over 4,500 g is also twice as great as in other infants. This means that the effect of birth weight on the incidence of CH generates a U-shaped curve [14]. The disease has also been observed in girls more frequently than in boys [14]. Hypothyroidism develops sporadically, and genetic factors play a minimal role in its development [15]. Premature infants have been found to be more vulnerable to the disease [16]. A maternal age over 40 years has been identified as a risk factor in some studies, as well as caesarian deliveries [17], but this hypothesis has been rejected by other reports [18]. Environmental factors, such as perchlorate, have a dose-dependent negative effect on the thyroid, and the toxins used in insecticides, such as organochlorine compounds, affect the incidence of CH [19-21]. Maternal goiter, pre-eclampsia, diabetes, sexually transmitted diseases, and diseases involving hypothyroidism increase the risk of neonatal hypothyroidism [12, 17,22]. Environmental factors increasing the incidence of CH include iodine deficiencies, exposure to high levels of iodine in the prenatal and postnatal period through mechanisms such as exposure to iodine and color imaging, maternal antibodies against the thyroid and pituitary glands resulting in dysfunction, dysfunctions in the pituitary feedback system, and mild disorders affecting thyroid hormone synthesis [23].

Due to the high prevalence of CH in Shadegan, Khuzestan Province, we conducted a study to determine the incidence of CH and to characterize the risk factors associated with CH. The practical purposes of this study include raising awareness of hypothyroidism, identifying high-risk groups, implementing screening for hypothyroidism, and providing a review of the relevant literature.

## MATERIALS AND METHODS

Initially, a descriptive study was conducted to investigate the incidence of CH from 2012 to 2014 in Shadegan, a county in Khuzestan Province with a population of over 159,503 people. The incidence of the disease according to age, sex, and season was also investigated. Furthermore, a case-control study was conducted to determine the relationship of CH with demographic, environmental, and medical factors. The process of diagnosing CH involved obtaining a 5-mL blood sample from the heels of 3-5-day-old infants using an auto-lancet and transferring the samples onto Guthrie filter papers. The blood samples were dried and sent to the provincial reference laboratory for assessment using an enzyme-linked immunosorbent assay. According to the guidelines of the Ministry of Health and Medical Education in Iran, levels of thyroid-stimulating hormone (TSH) >10 mU/L and a T4 level <6.5 mg/dL allow the diagnosis of CH. In addition, infants with a TSH level ≥20 mU/L were referred to a specialist for specific surveillance. Babies diagnosed with CH were considered cases. We also evaluated all live births within this time period. Infants who had TSH levels <5 mU/L obtained using the method outlined above or an intravenous test of TSH with results of 1.7 to 9.9 mU/L and a T4 level of 6.5 to 16.3 were considered normal. Controls were randomly selected from normal infants.

The following information was gathered for all participants: maternal age; the sex of the infant; infant weight; preterm birth; family history of genetic disease; whether the mother had a history of recurrent miscarriage; delivery method; the presence of certain diseases during pregnancy, such as maternal hypertension, diabetes, pre-eclampsia, thalassemia, asthma, iron deficiency anemia, mental disorders, sexually transmitted diseases, goiter, and maternal CH; and use of certain drugs during pregnancy, such as amiodarone, cytokines, dopamine, iodine, rifampin, and steroids. To increase the power of the study, more than one control was selected per case. A total of 457 controls were randomly selected from the city of Shadegan, 100 from Abadan, and 100 from Khorramshahr. In order to address recall bias, the medical records of patients and controls were used to collect the necessary information. The protocol also incorporated an analysis of environmental factors. Environmental health experts obtained three water samples from the Shadegan International Wetlands in the village of Sarakhyh on October 10, 2015 at 8:00 a.m., at a temperature of 21˚C and a humidity of 67%, and levels of nitrites, nitrate, chlorine, and iodide were assessed using a spectrophotometer (DR 2000; Hach, Loveland, CO, USA). Descriptive statistics, such as mean (standard deviation) and frequency (%), were used to describe demographic and clinical parameters, variables relating to the medical history of the patients’ mothers, and certain environmental factors. Statistical tests, such as the chi-square test and logistic regression modeling, were performed using SPSS version 18.0 (SPSS Inc., Chicago, IL, USA). A p-value <0.05 was considered to indicate statistical significance.

### Ethical considerations

The ethics committee of the Isfahan University of Medical Science approved this study (no. 394772).

## RESULTS

**Figure 1.**
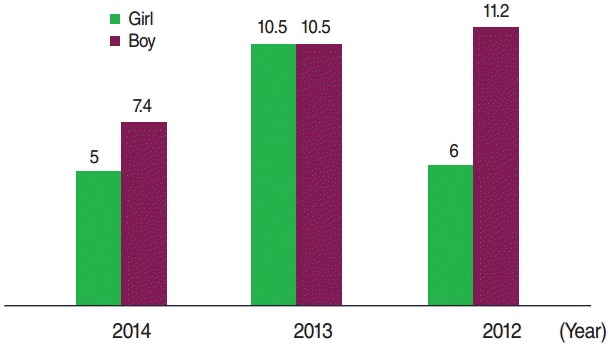
The incidence of congenital hypothyroidism in terms of sex per 1,000 live births

**Figure 2.**
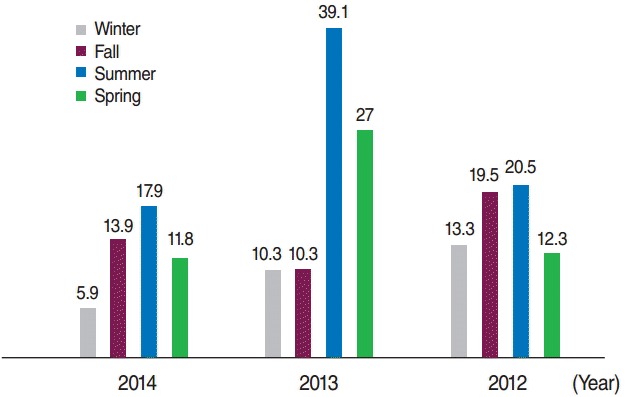
The incidence of congenital hypothyroidism in terms of season in 2012-2014 per thousand live births.

Of the 203 cases, 113 (55.7%) were boys, of whom 6 (2.9%) were premature. A total of 116 (57.1%) of the mothers of the cases had a history of consanguineous marriage. Further details regarding the characteristics of the patients and the controls are shown in Tables 1 and 2. The incidence of CH was 16.9, 21.5, and 12.6 per 1,000 live births in 2012, 2013, and 2014, respectively. The incidence of CH according to sex and the season when the infants were born is presented in Figures 1 and 2, respectively.

A significant relationship was found between a history of parental consanguinity and CH. The likelihood of CH in an infant born to consanguineous parents was 2.41 times greater than the likelihood of CH in infants born to parents without consanguinity (odds ratio [OR], 2.41; 95% confidence interval [CI], 1.65 to 3.53). A relationship was also found between where the baby was born and CH, with infants born in a rural area being 3.45 times more likely to have CH than infants born in urban areas (OR, 3.45; 95% CI, 2.29 to 5.20). No other variables showed a statistically significant relationship with CH (Table 3).

The chemical analyses of water samples from the Shadegan International Wetland showed that the average quantities of nitrate, nitrite, and iodide from three samples were 0.8 mg/L, 0.1 mg/L, and 0.24 mg/L, respectively, which were lower than the standard limits. No chlorine was detected. These results indicate that levels of nitrate, nitrite, iodide, and chlorine could not have played a role in the incidence of CH.

No relationships were found between CH and age, the sex of the infant, infant weight, preterm birth, family history of genetic disease, maternal history of recurrent miscarriage, or delivery method, as well as the presence of certain diseases during pregnancy (such as maternal hypertension, diabetes, pre-eclampsia, thalassemia, asthma, iron deficiency anemia, mental disorders, sexually transmitted diseases, goiter, or maternal CH) and the use of drugs during pregnancy, such as amiodarone, cytokines, dopamine, iodine, rifampin, or steroids.

## DISCUSSION

The incidence of CH was 16.9, 21.5, and 12.6 per 1,000 live births in 2012, 2013, and 2014, respectively. Studies conducted in Tehran, Isfahan, and Shiraz have found the average incidence of hypothyroidism in Iran to be one per 1,000 live births [6-8]. The incidence of congenital hypothyroidism is one per 4,500 live births in the US, one per 3,000 live births in Europe, and one per 5,700 live births in Japan, and the most important cause of congenital hypothyroidism has been found to be hereditary thyroid hormone deficiency [24].

This study demonstrated a relationship between CH and parental consanguinity. Parents in a consanguineous marriage were found to have a 2.41 times greater risk of having a child with CH than parents in a non-consanguineous marriage. Research conducted in Tehran showed that the incidence of CH in infants was 2 to 3 times greater than its average incidence in the world and that the high prevalence of the disease was significantly associated with consanguineous marriage. Thus, consanguineous marriage is a factor connected with the higher incidence of this disease in Iran. A case-control study carried out in Hamadan likewise showed a relationship between CH and parental consanguinity [25]. Hypothyroidism is sporadic and genetic factors have little role in its development [15]. In contrast, no significant association between consanguineous marriage and congenital hypothyroidism was found in a study conducted in Isfahan. In another study conducted in Tehran the OR of CH in patients whose parents had a consanguineous marriage was 2.7, giving further support to the hypothesis that consanguineous marriage influences the incidence of CH [26].

This study found a relationship between CH and urbanization. The OR associated with being born in an urban location for CH was 3.45, confirming the effect of urbanization on the high incidence of CH. Very few studies have been conducted on the geographical distribution of the incidence of CH [27]. In a study in Wales in the UK that analyzed 11 years of screening data, the incidence of CH in southern areas was lower than in northern areas, and the authors explained this discrepancy in terms of the higher population density in the northern areas of Wales [28]. In another study conducted in New York, non-significant differences were observed in the incidence of CH in New York compared to the incidence in other states, and the authors explained the presence of this discrepancy as being due to the high Asian population in locations with a high incidence of CH [29]. In another study performed in Iran using geographic information system mapping, the authors concluded that no particular geographical explanation accounted for the high incidence of CH in Iran, and that other potential explanations included non-environmental factors and factors related to the quality of screening programs [30]. The present study found no relationships between CH and age, sex of the infant, or the use of certain medications during pregnancy. The results of the Hamadan study are consistent with the results of our study in this regard [25].

The first major limitation of this study concerns the medical records of the pregnant mothers, and in particular, the fact that the nutrition column was not reliably filled out by the midwives and health workers. The second limitation is the fact that fish tissue could not be assessed for the presence of toxins and pesticides because no histology laboratory was available in the province. This is a relevant limitation because fish is a major part of the diet of the inhabitants of Shadegan, and toxins and pesticides in fish may be a risk factor for CH. The results of this study may be useful for preventive screening programs and can be applied to high-risk populations.

In summary, given the high incidence of CH in Shadegan County compared to other locations in Iran, the establishment of a CH screening program with an appropriate budget allocation is recommended. Consanguineous marriages increase the risk of CH, supporting the value of premarital counseling centers for educating couples about this issue. Urbanization also increases the risk of CH. Further studies should address the effect of factors such as maternal nutritional status and socioeconomic status on the incidence of CH.

## Figures and Tables

**Table 1.** Number of cases, live births, and cumulative incidence of congenital hypothyroidism per year in Shadegan

	2012	2013	2014
No. of cases	66	86	51
No. of live births	3,900	3,991	4,050
Cumulative incidence per 1,000 live births	16.9	21.5	12.6

**Table 2.** Characteristics of the participants in this study (2012-2014)

Characteristics		Cases	Controls
Sex	Male	113/203 (55.7)	314/657 (47.7)
	Female	90/203 (44.3)	343/657 (43.3)
Season	Spring	51/199 (25.6)	142/657 (21.6)
	Summer	76/199 (38.1)	230/657 (35.0)
	Fall	43/199 (21.6)	151/657 (22.9)
	Winter	29/199 (14.7)	134/657 (20.5)
Prematurity	Positive	6/201 (2.9)	18/657 (2.7)
Type of delivery	Caesarean	68/203 (33.4)	210/657 (31.9)
Location of birth	Urban	82/203 (40.4)	145/657 (22.0)
	Rural	121/203 (59.6)	512/657 (77.9)
History of consanguineous marriage	Positive	116/203 (57.1)	297/657 (45.2)
Pre-eclampsia	Positive	3/203 (1.4)	13/657 (1.9)
Diabetes	Positive	4/203 (1.9)	11/657 (1.9)
Anemia	Positive	56/203 (27.5)	223/657 (33.9)
Blood pressure	Positive	6/203 (2.9)	34/657 (5.1)
History of genetic disease	Positive	8/203 (3.9)	16/657 (2.4)
History of recurrent miscarriages	Positive	3/203 (1.4)	23/657 (3.5)
Amiodarone	Positive	0/203 (0.0)	0/657 (0.0)
	Negative	203/203 (100)	657/657 (100)
Cytokine	Positive	0/203 (0.0)	0/657 (0.0)
	Negative	203/203(100)	657/657 (100)
Dopamine	Positive	0/203 (0.0)	0/657 (0.0)
	Negative	203/203 (100)	657/657 (100)
Iodine	Positive	0/203 (0.0)	0/657 (0.0)
	Negative	203/203 (100)	657/657 (100)
Lithium	Positive	1/203 (0.5)	0/657 (0.0)
	Negative	202/203 (99.5)	657/657 (100)
Phenytoin	Positive	1/203 (0.5)	2/657 (0.3)
	Negative	202/203 (99.5)	655/657 (99.7)
Rifampin	Positive	1/203 (0.5)	1/657 (0.1)
	Negative	202/203 (99.5)	656/657 (99.8)
Steroids	Positive	2/203 (0.9)	1/657 (0.1)
	Negative	201/203 (99.1)	656/657 (99.8)
Hypertension	Positive	6/203 (3.0)	34/657 (5.0)
	Negative	197/203 (97.0)	623/657 (95.0)
History of genetic disease	Positive	8/203 (4.0)	16/657 (3.0)
	Negative	195/203 (96.0)	641/657 (97.0)
History of recurrent miscarriages	Positive	3/203 (0.2)	23/657 (4.0)
	Negative	200/203 (99.8)	643/657 (96.0)
Thalassemia	Positive	6/202 (3.0)	17/657 (2.5)
	Negative	196/202 (97.0)	640/657 (97.5)
Psychological disorder	Positive	1/203 (0.4)	2/657 (0.3)
	Negative	202/203 (99.6)	655/657 (99.7)
Asthma	Positive	2/203 (0.9)	8/657 (1.2)
	Negative	201/203 (99.1)	649/657 (98.8)
Sexually transmitted disease	Positive	1/203 (0.5)	8/657 (1.2)
	Negative	202/203 (99.5)	649/657 (98.8)
Goiter	Positive	1/203 (0.5)	5/657 (0.7)
	Negative	202/203 (99.5)	652/657 (99.3)
Maternal congenital hypothyroidism	Positive	0/203 (0.0)	5/657 (0.7)
	Negative	203/203 (100)	652/657 (99.3)

Values are presented as frequency (%).

**Table 3.** Results of a logistic regression analysis of the effects of participant characteristics on congenital hypothyroidism in Shadegan, Iran

Characteristics		OR (95% CI)	p-value
Sex	Male	1.00 (reference)	0.07
	Female	1.39 (0.97, 2.00)	
Season	Winter	1.00 (reference)	0.64
	Spring	1.25 (0.70, 2.22)	0.44
	Summer	1.37 (0.80, 2.32)	0.24
	Fall	1.10 (0.61, 1.96)	0.74
Type of delivery	Normal	1.11 (0.75, 1.65)	0.58
	Caesarean	1.00 (reference)	
History of consanguineous marriage	Yes	2.41 (1.65, 3.53)	< 0.001
	No	1.00 (reference)	
Age of mother		0.97 (0.94, 1.00)	0.13
Place of residence	Urban	3.45 (2.29, 5.20)	< 0.001
	Rural	1.00 (reference)	
Pre-eclampsia	Yes	1.95 (0.42, 8.90)	0.39
	No	1.00 (reference)	
Diabetes	Yes	1.29 (0.35, 4.67)	0.70
	No	1.00 (reference)	
Hypertension	Yes	0.55 (0.19, 1.60)	0.28
	No	1.00 (reference)	
Anemia	Yes	0.66 (0.43, 0.99)	0.07
	No	1.00 (reference)	
History of genetic disease	Yes	2.15 (0.85, 5.43)	0.10
	No	1.00 (reference)	
Recurrent miscarriages	Yes	0.23 (0.05, 1.09)	0.06
	No	1.00 (reference)	
Weight (g)	<2,000	2.20 (0.67, 7.20)	0.19
	>4,000	0.39 (0.13, 1.20)	0.10
	2,000-4,000	1.00 (reference)	0.10

OR, odds ratio; CI, confidence interval.
